# Angle Closure Scoring System (ACSS)-A Scoring System for Stratification of Angle Closure Disease

**DOI:** 10.1371/journal.pone.0160209

**Published:** 2016-10-27

**Authors:** Aparna Rao, Debananda Padhy, Sarada Sarangi, Gopinath Das

**Affiliations:** Glaucoma Service, LV Prasad Eye Institute, Patia, Bhubaneswar, India; Bascom Palmer Eye Institute, UNITED STATES

## Abstract

**Purpose:**

To evaluate the angle closure scoring system (ACSS) for stratifying primary angle course disease.

**Methods:**

This observational cross sectional institutional study included patients with primary open angle glaucoma suspects (n = 21) and primary angle closure disease (primary angle closure, PAC, n = 63 and primary angle course glaucoma, PACG, n = 58 (defined by International society of Geographical and Epidemiological Ophthalmology, ISGEO). Two independent examiners blinded to clinical details, graded good quality pre-laser goniophotographs of the patients incorporating quadrants of peripheral anterior synechieae (PAS), non-visibility of posterior trabecular meshwork (PTM) and blotchy pigments (ranging from 1–4 quadrants), iris configuration, angle recess (sum of above depicting ACSS_g_) and lens thickness/axial length ratio (LT/AL), cup disc ratio and baseline intraocular pressure (IOP) to give total score (ACSS_t_).

**Result:**

There were significant differences in ACSSg scores within the same ISGEO stage of PAC and PACG between eyes that required nil or >1medicines after laser iridotomy, p<0.001. The ACSSg was associated with need for >1 medicines in both PAC and PACG eyes, p<0.001. An ACSSg score>12 and 14 in PAC (odds ratio = 2.7(95% CI-1.7–5.9) and PACG (Odds ratio = 1.6(95%CI-1.19–2.2) predicted need for single medicines while ACSSg scores >14 and 19 predicted need for ≥2 medicines in PAC and PACG eyes, respectively. The LT/Al ratio, IOP score or cup disc score did not influence the need for medical treatment independently.

**Conclusion:**

The ACSS can be a useful clinical adjunct to the ISGEO system to predict need for medicines and prognosticate each stage more accurately.

## Introduction

Angle closure disease is a matrix of disease comprising of several stages proceeding from normal (yet potentially prone to damage) to gradual loss of optic nerve function.The biennial congress of the International Society for Geographical and Epidemiological Ophthalmology (ISGEO) group proposed a generalised classification system wherein the former stages without optic nerve dysfunction were termed as primary angle closure suspects (PACS) or primary angle closure (PAC), while those with established optic neuropathy, were termed as primary angle closure glaucoma (PACG).[1,2] This classification has gained worldwide acceptance owing to its applicability across epidemiological surveys. Yet, it falls short in various ways while prognosticating the disease based on this classification, which identifies only three stages, includes eyes with acute angle closure attacks into PAC eyes.[3–5] Our earlier study identified that roughly 1/3^rd^ of PAC eyes require medical treatment after laser peripheral iridotomy, LPI and roughly 1/3^rd^ of these require surgery for IOP control over long term follow up.[6] It is also known that roughly 1/3^rd^ of PACS progress to PAC and roughly a 1/3^rd^ of PAC eyes progress to PACG over long term follow up.[7–9]

It is not known what factors can truly prognosticate need for medical treatment after successful LPI or predict long term progression into the other stage. Current classification system fails to acknowledge this gradual transition of eyes from one stage to the other and does not provide any conclusive measure which can predict the long term prognosis in term of stability or progression or even need for medical treatment.[4,5] The role of this classification system for clinical use is also compounded by the fact that the transition from one stage to the other is rarely in steps and occurs gradually.[5,9] Older classification systems actually were inclusive in part of this fact when terms like creeping angle closure was used for eyes with progressive angle closure in a zipper fashion in some eyes.[10–17] Importance was also given to other anatomical attributes like axial length “(AL), lens thickness (LT) and anterior chamber depth (ACD), which are known risk factors in angle closure.[13–16] However this latter fact has not been conclusively proven to be the determining factor defining risk for closure in different ethnic populations. Therefore these earlier classification systems have gone out of use after the introduction of new classification system, which is now a standard way of classifying angle closure disease. Since this classifications system is applicable to wide usage across different ethnicities, an improvisation of the classification system identifying and incorporating risk factors in different ethnic populations, equipped to prognosticate the disease apart from classifying the disease is an essential requirement in routine clinical practise. We earlier proposed a simple scoring system by including gonioscopic and anatomical attributes to better indicate the overall prognosis of the eye applicable for any ethnic population.[5] This study evaluated the applicability of this scoring system in clinical use while incorporating anterior segment biometric parameters in angle closure disease.

## Methods

This observational cross sectional studyincluded newly diagnosed patients with primary open angle glaucoma (POAG) suspects (or controls) and primary angle closure disease (primary angle closure and primary angle course glaucoma) attending glaucoma services at our institute and seen by a single physician (APR). Data that were recorded included the best corrected visual acuity, spherical equivalent, gonioscopy by 4 mirror goniolens,applanation intraocular pressure (IOP) at the time of imaging, refractive error, fundus color and red free photographs, Humphrey visual fields (Carl Zeiss Meditec, 24–2, 10–2 and macular program)performed by single examiner blinded to patient. All procedures adhered to the tenets of the Declaration of Helsinki and the study was approved by the institutional review board of LV Prasad Eye Institute, Odisha, India. As institutional protocol, a written informed consent is taken from all patients undergoing examination or tests at the institute.

Gonioscopy was done by a single examiner (APR) using 4 mirror goniolens under standard lighting conditions and classified according to ISGEO classification system.[1,2] The treatment of the patient and further management of the patient was done as per guidelines. Details of medical treatment after laser peripheral iridotomy (LPI), need for surgery, presenting IOP and IOP on treatment, disc damage and visual field indices were recorded for each patient included into the study. Biometry was done by an independent examiner blinded to the clinical details to measure the AL, LT and ACD in each patient.

Slit lamp and gonio-photographs was imaged by a blinded technician (DP) using Visupac version 4.4.4 (FF 450 plus IR Carl Zeiss Ltd USA) photography system in non-indented and indented state using 4 mirror lens under low illumination, low and high magnification, 1mm slit size, avoiding light on the pupil. Images were taken for all quadrants and a video was taken in patients who could co-operate. Care was taken to avoid excess indentation as seen by visualisation of corneal folds. All images of good quality including all four quadrants were included for the study while those with poor quality images without discernible angle evaluation, corneal folds due to excess compression or any other corneal pathology, prior laser procedures or surgery were excluded. Images of adequate clarity showing the indented images in all 4 quadrants were now selected.

All images were now analysed separately by two examiners independently in a blinded manner without access to clinical details and the ISGEO classification, IOP, fundus or visual field details. The scoring system used by the examiners was as described previously. Briefly, this uses gonioscopic parameters including quadrants of peripheral anterior synechieae (PAS), non-visibility of posterior trabecular meshwork (PTM)and blotchy pigments (ranging from 1–4 quadrants), iris configuration, angle recess. This score,depicting gonioscopic parameters, now hereby referred to as sum score of gonioscopic parameters (ACSS_g_), was combined withlens thickness/axial length ratio (LT/AL), cup disc ratio and baseline intraocular pressure (IOP) to give total angle closure scoring system score (ACSS_t_), (S1 Table).

The ACSS_g_ and ACSS_t_ scores were now used for stratifying the patients into different subdivisions. The difference in scores in each ISGEO stage or subdivisionand the scores that predicted need for additional medical treatment after LPI was identified.

### Statistical Analysis

Intraclass co-efficient was used to evaluate agreement between two observers forthe scoring system for each eye. Normality was analysed using Shapiro-Wilk test while descriptive data were described as means and deviation for normally distributed and median and range for non-parametric variables.

Difference of scoring system in different strata within the same stage of ISGEO system were analysed and cut off for each stage was evaluated. Proportions were analysed using Chi-square test while agreement between observer for particular angle characteristics were evaluated using Bland-Altman Plots. Additional analysis of scoring system predicting need for medical treatment was evaluated by backward step-wise regression with significance set at 5%. Differences between eyes requiring medical treatment in each stage were analysed using one-way ANOVA with an alpha error set at <0.05.

## Results

Of 693 PAC and 1097 PACG seen during the period, 232 glaucoma patients with good quality goniophotographs, capturing clearly discernible details of all angle structures using a thin small beam with no light falling on the pupil, were obtained from hospital diagnostic database. Of these, 93 with previous laser/surgery during the time of photography or with incomplete photographs (all 4 quadrants images not available) were excluded. An additional 8 eyes were excluded due to concurrent posterior or anterior segment pathology causing secondary angle closure. A total of 121 goniophotographs of patients with primary angle closure disease naive to medical or laser/surgical treatment with good quality images of all 4 quadrants were finally chosen for analysis.

Of these, 63 eyes with PAC and 58 PACG, classified clinically according to ISGEO stages, were identified which fulfilled all inclusion criteria including complete data. The goniophotographs of these patients were compared with 21 eyes with POAG suspects (that were naïve to medicines or any form of laser or surgery) and eyes with suspect discs. The ICC for quadrants of PAS [0.74 (0.68 to 0.84)], PTM [0.81 (0.74 to 0.86)] and blotchy pigments [0.92 (0.89 to 0.94)] was excellent between the two observers (ophthalmologist and optometrist involved (APR and DP, respectively). S1 and S2 Figs show the Bland-Altman plot for quadrants of PAS and blotchy pigments between the two observers.

Table 1 gives the baseline demographic characteristics among PAC, PACG eyes with 21 controls. While all parameters including PASquadrants, IOP and other differed significantly, the LT/AL ratio did not differ significantly between PAC and PACG eyes, p = 0.1 though they were significantly different compared to controls. The sum scores of PAC and PACG differed significantly between PAC and PACG, Table 1.

**Table 1:** Baseline characteristics of patients with primary angle closure, primary angle closure glaucoma and controls included in the study.

Scoring parameters	PACG	PAC	Controls	P value Anova
Baseline IOP	27±12.7	16±5.2	15±4.5	<0.001
Baseline medicines	1±1.1	0.2±0.5	0	<0.001
Quadrants of PAS	2±0.9	1±0.9	0.3±0.5	<0.001
PTM non visibility	2.5±1.06	1.5±0.8	0.4±0.6	<0.001
Quadrants of blotchy pigment	3±0.9	1.7±0.9	0.9±0.2	<0.001
Angle Recess	3±1.06	2±0.9	2±1.4	<0.001
Iris configuration	3±1.1	2±1.6	2±1.2	0.001
Sum Gonioscopic parametersACSSg score	14±4.1	9±3.2	7±2.8	<0.001
Sum gonioscopic+LT/ALratio+IOP+Cup disc ratio-ACSSt score	18±5.4	14±3.9	8±2.1	0.013

IOP-intraocular pressure; Medicines-anti-glaucoma medications; PAS-peripheral anterior synechiae

PTM-Posterior trabecular meshwork; LT/AL- lens thickness/axial length ratio

The PAC and PACG was now stratified according to gonioscopic sum scoresACSSg<25^th^ (strata 1)25-75^th^ (strata 2) and >75^th^(strata 3) percentile. Table 2 gives the difference in gonioscopic parameters in each of these strata or tier showing significant differences between strata 3 and earlier tiers in PAC and PACG eyes. Interestingly, there was overlap between PACG eyes in strata 1 or 2 with PAC eyes in strata 3 with significant overlap of gonioscopic parameters including quadrants of PAS, blotchy pigments or quadrants of non-visibility of PTM, p = 0.0001 for each, Table 2.

**Table 2:** Stratification of eyes with primary angle closure based on <25^th^ (strata 1), 25-75^th^ (Strata 2) and >75^th^ (Strata 3) percentile of sum scores of gonioscopic parameters (ACSSg).

Primary angle closure				
Variables	Strata 1 N = 21 Mean±SD	Strata2 N = 29 Mean±SD	Strata 3 N = 13 Mean±SD	P value Anova
Age	56±9.6	55±10.7	51±9.1	0.62
IOP (mean, range)	15±2.8, 11–21	16±3.5	19±9.2	0.74
Quadrants of PAS, (Range)	0.6±0.7, 0–2	1±0.8, 0–3	2±0.8, 2–4	0.0001
Quadrants of Blotchy pigments,(Range)	• 1.2±0.6 • 0–2	• 1.7±0.7 • 0–3	• 2.7±0.8 • 2–4	0.0001
Quadrants of PTM non-visibility,(Range)	• 1±0.5 • 0–2	• 1±0.6 • 1–3	• 2.5±0.8 • 2–4	0.0001
Angle Recess score	1.4±0.6	2±0.7	3±0.8	0.0001
Iris configuration score	1.5±0.7	2.6±1	3.6±0.6	0.0001
Medicines	0	• 2 (6.8%) • 27 required 0medicines • 2 required 1 medicines	• 10 (76.9%) • 3 required 0medicines • 6 required 1 medicines • 4 required 2 medicines	<0.0001-chisquare
Primary angle closure glaucoma				
Variables	Strata 1 N = 19	Strata2 N = 32	Strata 3 N = 7	P value Anova
Age (years)	64±10.1	61±13.03	56±10.7	0.06
IOP (mm Hg)	17±3.3	30±13.03	36±12.7	0.001
Quadrants of PAS, (range)	• 1.5±0.61 • –3	• 2.7±0.71 • –4	• 3.8±0.3 • 3–4	0.0001
Quadrants of Blotchy pigments, (range)	• 2.2±0.9 • 1–4	• 3±0.7 • 1–4	• 3.8±0.3 • 3–4	0.0001
Quadrants of PTM non visibility,(range)	• 1±0.9 • 1–4	• 2.8±0.6 • 2–4	• 3.8±0.1 • 3–4	0.0001
Angle Recess score	2±0.9	3.5±0.6	3.8±0.3	0.0001
Iris configuration score	1.9±1.1	3.9±0.2	4±0.2	0.0001
Medicines	• 9 (47.3%) • 10 required 0medicines • 6 required 1 • 3 required 2 medicines • 0 required 3 and 4 medicines	• 22 (68.75%) • 10 required 0medicines • 10- required 1 medicines • 10 required 2 medicines • 1 required 3 • 1 required 4 medicines	• 7 (100%)0 required 0medicines2 required 1medicines1 required 2 medicines2 required 3 medicines2 required 4 medicines	0.0001

IOP-intraocular pressure; Medicines-anti-glaucoma medications; PAS-peripheral anterior synechiae; PTM-Posterior trabecular meshwork

**Fig 1:**
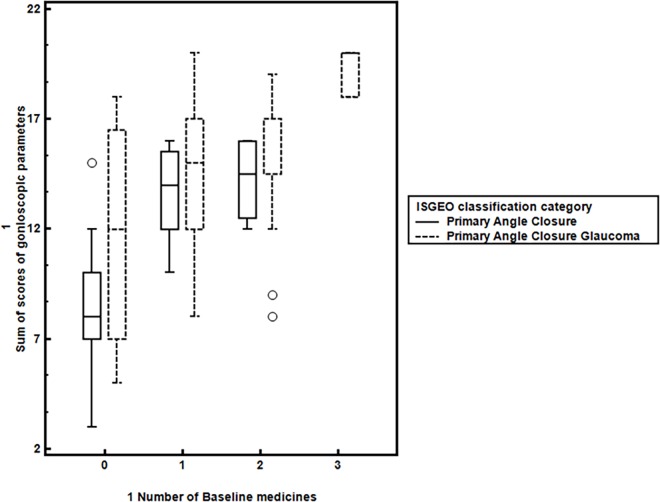
Comparison of sum of gonioscopic scores (ACSSg) with number of baseline medicines required in eyes with primary angle closure (PAC) and primary angle closure glaucoma (PACG)-circles represent outliers.

**Fig 2:**
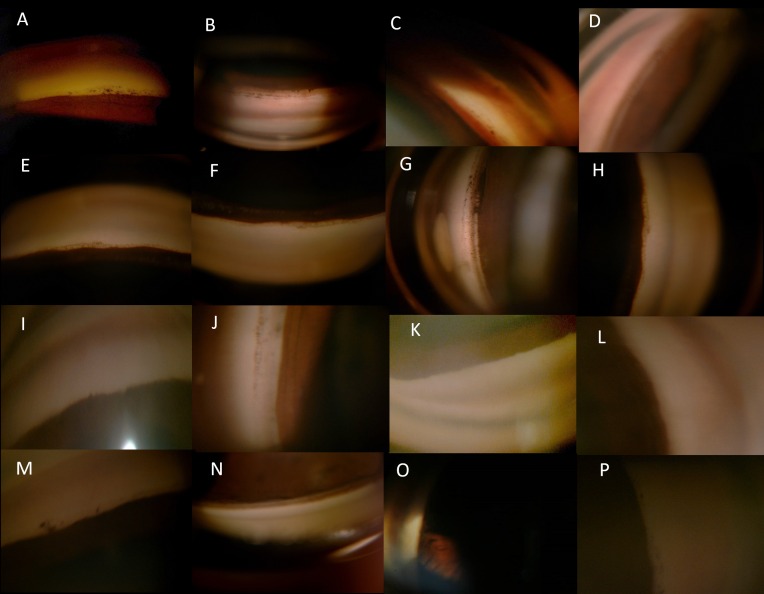
Comparison of 4 quadrant (from left to right superior, inferior, temporal and nasal in that order) gonioscopic features of eyes with primary angle closure requiring 0 medicines (a-d) with that requiring 1 medicines (E, F, G and H) or 2 medicines (I, J, K & L and M,N, O &P) showing increasing quadrants of peripheral anterior synecheiae/blotchy pigments/non-visibility of posterior trabecular meshwork and narrower recess with increasing need for number medicines.

**Fig 3:**
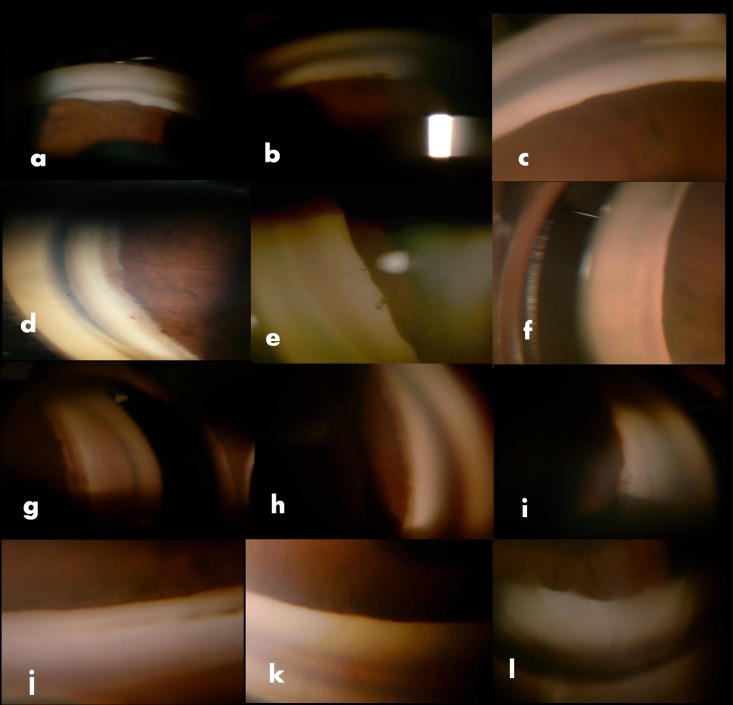
Comparison of 4 quadrant (from above to below superior, nasal, temporal and inferior in that order) gonioscopic features of eyes with primary angle closure glaucoma requiring 0 medicines (group 1-a,d, g and i) with that requiring 1 medicines (group 2-b, e, h and k) or >2 medicines (group 3-c, f, i, l)showing definite differences in gonioscopic features among eyes with different number of medicines, especially group 1 and group 3.

Evaluating the difference further in ISGEO tier of PAC eyes, 8 required 1 or 2 medicines with none of the eyes requiring >2 medicines for IOP control. We found significant difference in those requiring no medicine after LPI versus those that required medicines for IOP control, Tables 3 and 4, Fig 1, p<0.001. Specifically, we tried to evaluate differences in PAC eyes requiring one or more medicines. Table 3 shows the scores in PAC eyes requiring one or more than one medicine showing significant difference between those requiring 1 or >1 medicines as compared to those requiring no medicines, p<0.001, though the scores for eyes requiring either 1 or two medicines were not statistically different. The baseline IOP, quadrants of PAS in each of these subgroups was not significantly different among eyes that required 1 or no medicines, Table 4. On univariate analysis of each of the gonioscopic parameters, quadrants of PTM non-visibility was significantly associated with >1 medicines while baseline IOP, age, angle recess and iris configuration were not associated significantly with need for medicines. Interestingly, eyes which required 1 or 2 medicines had comparable quadrants of PTM non-visibility with no significant difference in angle recess or iris configuration in these eyes. Multivariate analysis showed that the sum ACSSg was independently associated with need for >1 or 2medicines, p<0.001 while each gonioscopic feature did not independently influence the need for medicines, Figs 2 & 3, S2 Table. Combining the scores for LT/AL ratio showed only a marginal increased risk for need for medicines, (S3 Table).

**Table 3:** Differences in sum scores of gonioscopic parameters (ACSSg) eyes with primary angle closure (PAC) and primary angle closure glaucoma (PACG) eyes requiring medications after laser iridotomy.

	PAC, Mean±SD, N	PACG, Mean±SD, N	Controls,Mean±SD, N	P value
Gonioscopic sum scores in eyes requiring 0 medicines	8±2.3, 51	12±4.6 ,20	6±2.7, NA	0.01
Gonioscopic sum scores in eyes requiring 1 medicines	13±2.1, 8	14±3.1, 18	NA, 0	0.001
Gonioscopic sum scores in eyes requiring 2 medicines	14±2.06,4	15±3.5 15	NA,0	0.01
Gonioscopic sum scores in eyes requiring >2medicines	NA,0	19±1.09, 5	NA,0	NA

**Table 4:** Comparison of clinical and gonioscopic parameters in primary angle closure or primary angle closure glaucoma eyes requiring different number of anti-glaucoma medications after laser iridotomy.

Primary angle closure				
Variables	Group 1 (0 medicines) N = 51	Group 2(1 medicines) N = 8	Group 3(2 medicines) N = 4	P value Anova
Age (years)	52±10.3	51±6.2	49±10.7	0.63
IOP (mm Hg)	15±3.1	20±10.8	18±6.7	0.31 (0 &1)
Quadrants of PAS	0.9±0.8	2±0.7	2±1.3	0.001
Quadrants of Blotchy pigments	1±0.7	3±0.5	3±0.8	0.0005
PTM non visibility	1±0.6	2±0.9	2±0.9	0.002
Angle Recess	2±0.8	2±0.7	3±0.5	0.004
Iris configuration	2±0.9	3±1.06	4±0.0	0.001
Primary angle closure glaucoma				
Variables	Group 1 (0 medicines), N = 20	Group 2 (1–2 medicines), N = 33	Group 3(>2 medicines), N = 5	P value, Anova
Age (years)	59±12.5	64±10.2	53±15.9	0.17
IOP (mm Hg)	26±13.5	25±11.7	39±11.08	0.06
Quadrants of PAS	2±0.9	2±0.9	3.8±0.4	0.002
Quadrants of Blotchy pigments	2±1.3	3±0.6	4±0	0.02
PTM non visibility	2±1.1	3±0.9	3±0.8	0.03
Angle Recess	2±1.1	3±0.9	4±0.4	0.03
Iris configuration	2±1.3	3±1.0	4±0	0.02

IOP-intraocular pressure; Medicines-anti-glaucoma medications; PAS-peripheral anterior synechiae

PTM-Posterior trabecular meshwork

Evaluating differences in PACG eyes requiring different number of medications after iridotomy, the sum scores of gonioscopic parameters was significantly greater in PACG eyes requiring >2 medicines, Table 3. Since those requiring 1–2 medicines or 3–4 medicines were comparable, we grouped these eyes together into two groups respectively, Table 4. There were significant differences in gonioscopic parameters withmaximal differences in eyes requiring more than 2 medicines for IOP control, Tables 3 & 4. Evaluating each parameter separately, >2 quadrants of PAS and blotchy pigments predicted need for >2 medicines for IOP control in these eyes, Table 4 and S2 Table. Only the sum ACSSg score of gonioscopic parameters was significantly associated on multivariate analysis, S2 Table, S3 Fig and S4 Fig.

The sum ACSSg>12 and 14 in PAC (odds ratio = 2.7(95% CI-1.7–5.9) and PACG(Odds ratio = 1.6(95%CI-1.19–2.2) predicted need for single medicines while sum ACSSg scores >14 and 19 predicted need for 2 or more medicines in PAC and PACG eyes, respectively, S2 Table.

Evaluating now the result of adding LT/AL ratio and baseline IOP scores, there was minimal change in total ACSStscores as compared to sum scores of gonioscopic parameters alone (S3 Table). Univariate analysis showed the LT/Al ratio,IOP scoreorcup disc score did not influence the need for medical treatment independently in PAC or PACG eyes.

## Discussion

In this study, definite difference in scores of angle scoreswere seen between PAC and PACG eyes as well as between eyes requiring 1 or more medicines within the same ISGEO group of PAC and PACG eyes. This reflects that the current staging system of PAC and PACG is inadequate in appropriately stratifying or prognosticating eyes since there may be more stages in each ISGEO stage of PAC and PACG during the evolution or progression of angle closure disease. The sum ACSSg was independently associated with need for >1 medicines with minimal change with addition of other parameters.

Caveats of the pre-existing scoring system: Our scoring system not only identified PACG eyes requiring one or two medicines but also identified significant overlap of scores between PAC eyes at one stage and PACG eyes in the preceding stage. This reflects non-linear progression of PAC and PACG eyes at each stage and reiterates the importance of scoring systems for prognosticating PAC eyes or PACG eyes after initial definitive treatment. Currently, the ISGEO system, globally used in epidemiological surveys, is also used for clinical practise which has been known to be inappropriate on several fronts. [5] Though several studies have contributed significantly to the understanding angle closure disease, a clinically adaptable classification system prognosticating disease at each stage is yet to emerge.[3,4] The primary tissue of injury, the trabecular meshwork, still eludes any classification as to the extent of damage in each stage, which is presumed to be indirectly inferred from previous iridotrabecular contact.[13,14,16,17] Treatment of all PAC or PACG eyes with iridotomy fails to control IOP in all cases with a third progressing to the next stage or requiring additional medical treatment.[6–9, 18–24] Differences in outcomes after LPI in the same stages (using the ISGEO system) across studies suggests the underlying differences of extent to damage in different stages in their evolution to glaucoma, which cannot by any means be captured or differentiated by the current classification system. Different systems used to define different stages of glaucoma and lack of adequate pre-operative stratification may be the cause for disparity in results of combined vs cataract surgery in PACG since level of baseline TM damage may be different in these studies. The ISGEO system accepts that >270 non-visibility of PTM is an arbitrary way of representing angle damage. Other angle features like iris or lens thickness or role of other risk factors like age and axial length may also determine which eyes on therapy would require surgery.[13–15,25,26] Possibly a detailed description of the changes over time of angle characteristics as used in this scoring system is understandably a better way of identifying angle closure eyes at risk for progression.

Importance of adequate baseline staging: It is accepted that anatomical factors play an important role in determining the “crowdability” of the angle tough the results across ethnic populations have not been concordant.[13,25,26] Mapstone hypothesised that anterior movement of lens does not increase the pupillary block force directly but instead causes favourable conditions for irido-corneal contact.[27] This explains the benefit of lens extraction in PAC eyes without permanent trabecular damage with repeated episodes of irido-corneal contact.[28,29] Newer imaging techniques have now shifted the focus on the lens vault rather than axial lens thickness.[29,30] The importance of iris configuration has been renewed with the use of modern angle imaging systems with due recognition of varied course of eyes with plateau iris after LPI.[29–34] We used the ratio of lens thickness/axial length to reflect the dynamic interplay between the two factors which obviates undue importance to absolute values of each parameter in each stage and therefore is applicable in different ethnic populations.

There is considerable debate of the ISGEO system failing to incorporate APAC eyes into a separate group.[35,36] Patients with silent episodic angle closure (ex PAC eyes with prior angle closure) or those in chronic glaucoma stage would both be considered to be part of the same tier as per this ISGEO system.[5] The revised scoring system would therefore consider sudden impact of changes in angle anatomy, the role of lens/axial length and angle damage apart from recognizing plateau iris, combined mechanism glaucoma as different factors that evolve over time to cause progressive damage driving targeted (lens or iris specific) therapies.

This study was limited to a subset of patients seen at a single tertiary centre. Since we wanted to evaluate the use of this scoring system in established PACD, we included PAC and PACG eyes in this study. Dynamic changes under physiologic conditions cannot be incorporated into this scoring system which is based on clinical gonioscopic documentation during office visits. Nevertheless, this scoring system can be a useful addendum to the ISGEO system of PACD classification which may help prognosticate each stage of PAC and PACG quantitatively and accurately.

## Supporting Information

S1 Fig Bland Altman Plot showing agreement for scoring peripheral anterior synechieae between observers using the gonioscopic angle closure scoring system (ACSS). (TIF)

S2 Fig Bland Altman Plot showing agreement for scoring blotchy pigments in the angle between observers using the gonioscopic angle closure scoring system (ACSS). (TIF)

S3 Fig Association of sum of modified gonioscopic score (ACSSg) with number of medicines in eyes with primary angle closure. (TIF)

S4 Fig Association of sum of modified gonioscopic score (ACSSg) with number of medicines in eyes with primary angle closure glaucoma. (TIF)

S1 Table Modified angle closure scoring system (ACSS) for staging angle closure disease including gonioscopic (ACSSg) and other parameters (ACSSt) (PDF)

S2 Table Regression statistics predicting need for medical treatment in PAC and PACG eyes (PDF)

S3 Table Total sum scores using angle closure scoring system (ACSS) of gonioscopic parameters (ACSSg) with Lens thickness/axial length ratio, cup disc ratio and baseline intraocular pressure (ACSSt) in eyes requiring different number of medicines after laser iridotomy. (PDF)
