# Supplementary figures and images for: Angle Closure Scoring System (ACSS)-A Scoring System for Stratification of Angle Closure Disease
Source: PLoS One. 2016 Oct 27;11(10):e0160209. doi: 10.1371/journal.pone.0160209 (PMC5082952; doi:10.1371/journal.pone.0160209)

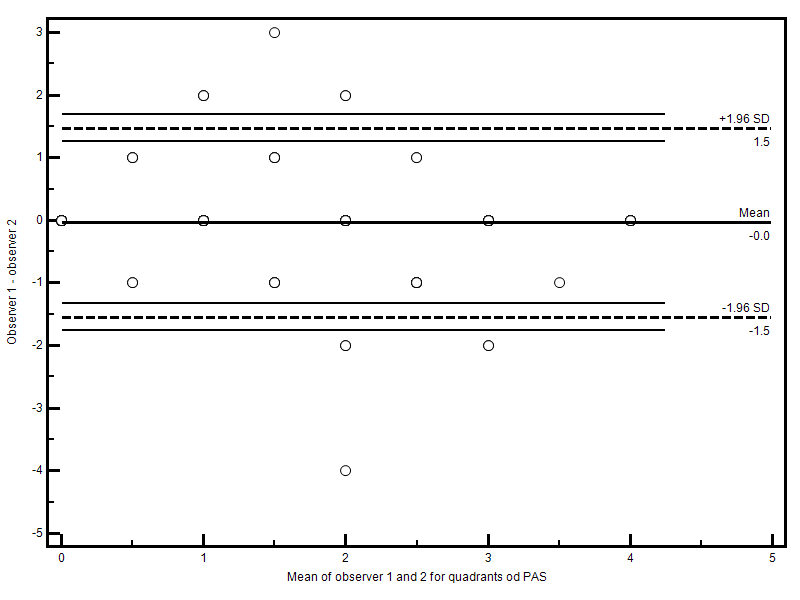

Supplement: S1 Fig — (TIF) [file pone.0160209.s001.tif]

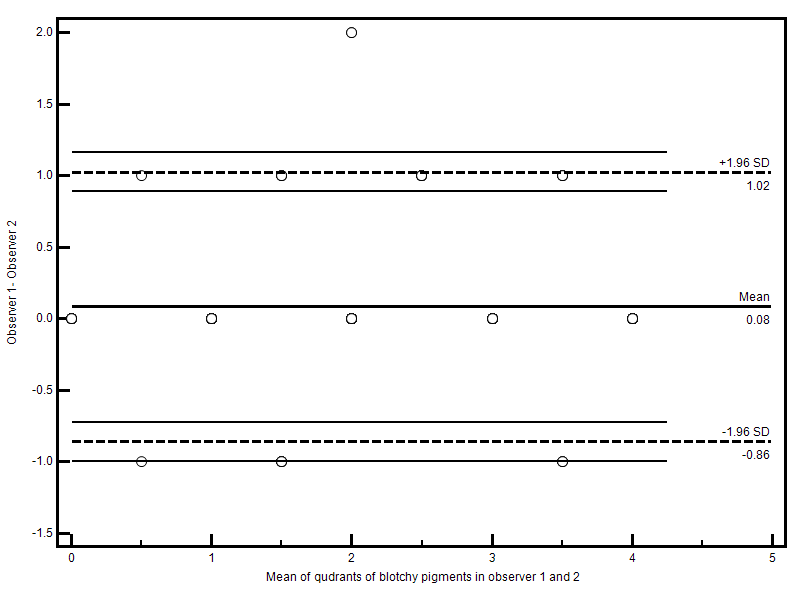

Supplement: S2 Fig — (TIF) [file pone.0160209.s002.tif]

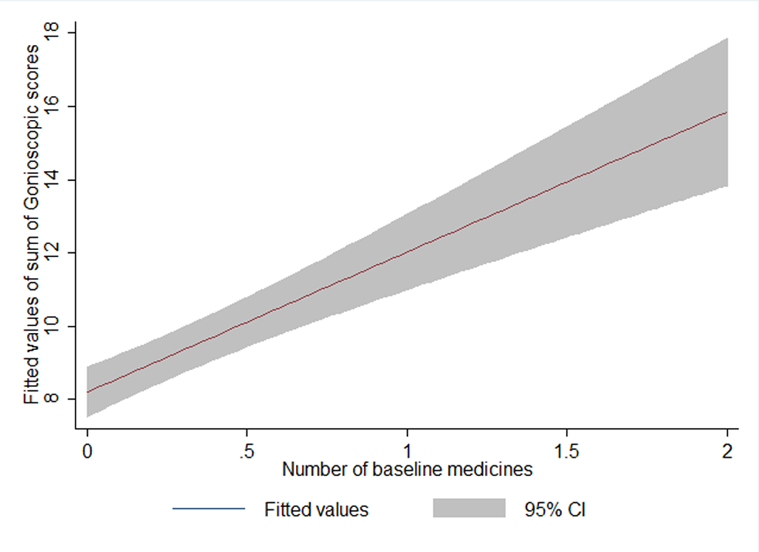

Supplement: S3 Fig — (TIF) [file pone.0160209.s003.tif]

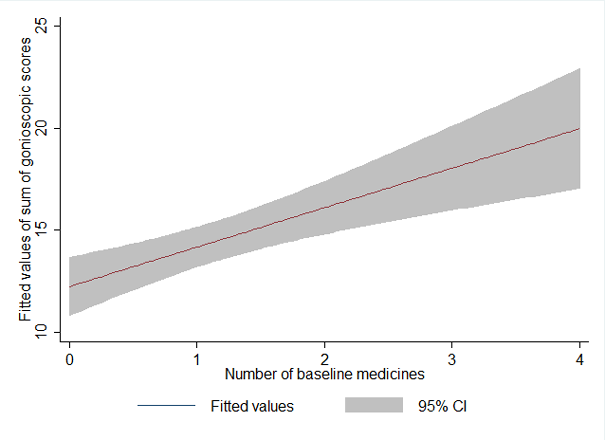

Supplement: S4 Fig — (TIF) [file pone.0160209.s004.tif]
