# Supplementary material for: Angle Closure Scoring System (ACSS)-A Scoring System for Stratification of Angle Closure Disease
Source: PLoS One. 2016 Oct 27;11(10):e0160209. doi: 10.1371/journal.pone.0160209 (PMC5082952; doi:10.1371/journal.pone.0160209)
Supplement: S1 Table — (PDF) [file pone.0160209.s005.pdf]

Supplemental Table 1: Modified angle closure scoring system (ACSS) for staging angle closure disease including gonioscopic (ACSSg) and other parameters (ACSSt)

	Angle closure scoring system (ACSS) Quadrant on gonioscopy/ severity of damage*			
	1	2	3	4
ACSSg				
PTM non-visibility	1Q	2Q	3Q	4Q
PAS/Goniosynechieae	1Q	2Q	3Q	4Q
Blotchy pigments	1Q	2Q	3Q	4Q
Angle Recess	>30^0	15-30^0	5-15^0	<5^0
Iris configuration/bowing	Concave	regular	PIC	Convex
ACSSt=ACSSg+below				
Cup disc ratio	<0.3	0.3-0.5	0.5-0.7	>0.7
IOP	<21	21-25	25-30	>30
LT/AL ratio	≤0.19	0.19-0.195	0.196-0.199	>0.199

Q-Quadrant of involvement; *-0 quadrants scored 0; IOP-Intraocular pressure; LT/AL-lens thickness/axial length ratio; PAS-peripheral anterior synecheiae; PTM-Posterior trabecular meshwork
