# Supplementary material for: Angle Closure Scoring System (ACSS)-A Scoring System for Stratification of Angle Closure Disease
Source: PLoS One. 2016 Oct 27;11(10):e0160209. doi: 10.1371/journal.pone.0160209 (PMC5082952; doi:10.1371/journal.pone.0160209)
Supplement: S2 Table — (PDF) [file pone.0160209.s006.pdf]

Supplemental Table 2: Regression statistics predicting need for medical treatment in primary angle closure (PAC) and primary angle closure glaucoma (PACG) eyes

	PAC			PACG		
1 medicines	Β	P value	Odds Ratio	β	P value	Odds Ratio
	0.9	0.012	2.7 (1.2-5.9)	0.4	0.002	1.6(1.2-2.2)
2 medicines						
	0.8	0.02	3.2 (1.8-4.6)	0.5	0.002	1.9 (1.3-5.3)
