# Supplementary material for: Angle Closure Scoring System (ACSS)-A Scoring System for Stratification of Angle Closure Disease
Source: PLoS One. 2016 Oct 27;11(10):e0160209. doi: 10.1371/journal.pone.0160209 (PMC5082952; doi:10.1371/journal.pone.0160209)
Supplement: S3 Table — (PDF) [file pone.0160209.s007.pdf]

Supplemental Table 3: Total sum scores using angle closure scoring system (ACSS)of gonioscopic parameters (ACSSg) with lens thickness/axial length ratio, cup disc ratio and baseline intraocular pressure (ACSSt)in eyes requiring different number of medicines after laser iridotomy.

Primary angle closure				
	Baseline Medicines=0	Baseline medicines=1	Baseline medicines=2	P value
IOP scores	1±0.2	2±0.2	3±0.5	0.008
LT/AL ratio scores	2±1.1	3±0.9	3±1.1	0.08
Cup disc ratio score	1±0.3	1±0.6	1±1.9	0.06
Sum score (ACSSt)	11±3.07	17±2.5	19±2.9	0.0001
Primary angle closure glaucoma				
	Baseline Medicines=0	Baseline medicines=1 and 2	Baseline medicines>2	
IOP scores	2±1.3	2±1.1	4±0.4	0.1
LT/AL ratio scores	2±1.04	2±0.9	3±0.5	0.3
Cup disc ratio score	2±0.5	2±1.2	2±2.1	0.6
Sum score ACSSt	17±6.07	19±4.5	25±1.5	0.007

IOP-intraocular pressure; Medicines-anti-glaucoma medications; PAS-peripheral anterior synechiae;

PTM-Posterior trabecular meshwork; LT/AL-Lens thickness/axial length ratio
